# A 360° Approach to Personalize Lifestyle Treatment in Primary Care for People With Type 2 Diabetes: Feasibility Study

**DOI:** 10.2196/57312

**Published:** 2024-12-04

**Authors:** Zeena Harakeh, Iris de Hoogh, Anne-Margreeth Krijger-Dijkema, Susanne Berbée, Gino Kalkman, Pepijn van Empelen, Wilma Otten

**Affiliations:** 1 Department of Child Health TNO, Netherlands Organization for Applied Scientific Research Leiden Netherlands; 2 Department of Microbiology and Systems Biology TNO, Netherlands Organization for Applied Scientific Research Leiden Netherlands; 3 Department of Internal Medicine Leiden University Medical Center Leiden Netherlands; 4 Academic Pharmacy Stevenshof Leiden Netherlands; 5 Health Center Stevenshof Leiden Netherlands; 6 Department of Risk Analysis for Products in Development TNO, Netherlands Organization for Applied Scientific Research Utrecht Netherlands; 7 Department of Sustainable Productivity and Employability TNO, Netherlands Organization for Applied Scientific Research Leiden Netherlands

**Keywords:** type 2 diabetes, diagnostic tool, holistic approach, personalized treatment, shared decision-making, health professionals, intervention, feasibility study, primary care

## Abstract

**Background:**

Given the multifactorial nature of type 2 diabetes (T2D), health care for this condition would benefit from a holistic approach and multidisciplinary consultation. To address this, we developed the web-based 360-degree (360°) diagnostic tool, which assesses 4 key domains: “body” (physical health parameters), “thinking and feeling” (eg, mental health and stress), “behavior” (lifestyle factors), and “environment” (eg, work and housing conditions).

**Objective:**

This work examines the acceptability, implementation, and potential effects of the 360° diagnostic tool and subsequent tailored treatment (360° approach) in a 6-month intervention and feasibility study conducted in standard primary health care settings in the Netherlands.

**Methods:**

A single-group design with baseline, 3-month, and 6-month follow-ups was used. A total of 15 people with T2D and their health care providers from 2 practices participated in a 6-month intervention, which included the 360° diagnosis, tailored treatment, and both individual and group consultations. The 360° diagnosis involved clinical measurements for the “body” domain and self-reports for the “thinking and feeling,” “behavior,” and “environment” domains. After multidisciplinary consultations involving the general practitioner, pharmacist, nurse practitioner (NP), and dietitian, the NP and dietitian provided tailored advice, lifestyle treatment, and ongoing support. At the end of the intervention, face-to-face semistructured interviews were conducted with health care professionals (n=6) and participants (n=13) to assess the acceptability and implementation of the 360° approach in primary health care. Additionally, data from 14 participants on the “thinking and feeling” and “behavior” domains at baseline, 3 months, and 6 months were analyzed to assess changes over time.

**Results:**

The semistructured interviews revealed that both participants with T2D and health care professionals were generally positive about various aspects of the 360° approach, including onboarding, data collection with the 360° diagnosis, consultations and advice from the NP and dietitian, the visual representation of parameters in the profile wheel, counseling during the intervention (including professional collaboration), and the group meetings. The interviews also identified factors that promoted or hindered the implementation of the 360° approach. Promoting factors included (1) the care, attention, support, and experience of professionals; (2) the multidisciplinary team; (3) social support; and (4) the experience of positive health effects. Hindering factors included (1) too much information, (2) survey-related issues, and (3) time-consuming counseling. In terms of effects over time, improvements were observed at 3 months in mental health, diabetes-related problems, and fast-food consumption. At 6 months, there was a reduction in perceived stress and fast-food consumption. Additionally, fruit intake decreased at both 3 and 6 months.

**Conclusions:**

Our findings suggest that the 360° approach is acceptable to both people with T2D and health care professionals, implementable, and potentially effective in fostering positive health changes. Overall, it appears feasible to implement the 360° approach in standard primary health care.

**Trial Registration:**

Netherlands Trial Register NL-7509/NL-OMON45788; https://onderzoekmetmensen.nl/nl/trial/45788

## Introduction

### Prior Work

Type 2 diabetes (T2D) is a multifactorial, chronic disease influenced not only by lifestyle and physical health factors but also by mental health and environmental factors. These factors are considered risk factors and also shape how individuals experience and manage their T2D [1-5]. In the Netherlands, people with T2D are primarily cared for by nurse practitioners (NPs) under the supervision of general practitioners (GPs) in primary health care [6]. Since 2015, Dutch health care providers, following established guidelines, have prioritized lifestyle changes as the initial focus of treatment. If these efforts yield insufficient results, medication is then prescribed [7]. In other words, the current focus in primary health care is primarily on physical health (eg, medication) and lifestyle factors such as smoking, diet, weight control, and exercise, while other important factors are often overlooked. Consequently, care for individuals with T2D could be enhanced by adopting a more holistic and personalized approach. This includes multidisciplinary consultations (eg, involving GPs, NPs, dietitians, physiotherapists, and pharmacists) and more frequent interactions between patients and health care professionals [8]. There are both national and international web-based tools that take a holistic approach, such as the “web diagram,” which focuses on positive health and primary health care and incorporates self-reported survey instruments [9]. However, given the multifactorial nature of T2D, a holistic approach would also benefit from incorporating clinical measurements of biomarkers to more comprehensively assess physical health factors. To address this, we developed the web-based 360-degree (360°) diagnostic tool to gain a comprehensive perspective on the health status of individuals with T2D, integrating both self-reports and biomarker data. This tool is designed to support personalized, patient-centered treatment [10]. Its objectives are improving shared treatment decision-making between health care professionals and individuals with T2D in primary health care, as well as enhancing self-management, empowerment, and informed decision-making for patients. A pilot evaluation of the tool found that health care professionals and individuals with T2D considered it relevant, clear, and practical [10].

### Objectives

In this study, we assessed the feasibility of implementing the 360° diagnostic tool in primary health care in the Netherlands. This paper presents the results of the feasibility study, focusing on the acceptability, implementation, and potential effects of the 360° approach within standard primary health care. Addressing these 3 outcomes provides insights into (1) whether individuals with T2D and health care professionals respond positively to the 360° approach; (2) its feasibility for implementation as planned by identifying advantages, disadvantages, and areas for improvement; and (3) the potential health outcomes (ie, measured parameters) for individuals with T2D over time [11]. As this is a feasibility study, the latter can only be tested to a limited extent (limited-efficacy testing). Nonetheless, these insights into the 360° approach offer valuable information about which components are essential and support the integration of this approach into standard primary health care.

## Methods

### Ethical Considerations

The study protocol was approved by the Medical Ethics Committee Brabant (NL67846.028.18) on January 8, 2019, and was conducted in accordance with the Declaration of Helsinki and good clinical practice guidelines. The study is registered in the Netherlands Trial Register (NL-7509; NL-OMON45788). A detailed description of the study methods is available in De Hoogh et al [12]. All professionals and participants provided written informed consent and granted permission to record the interviews.

### The 360° Approach and Study Design

**Figure 1:**
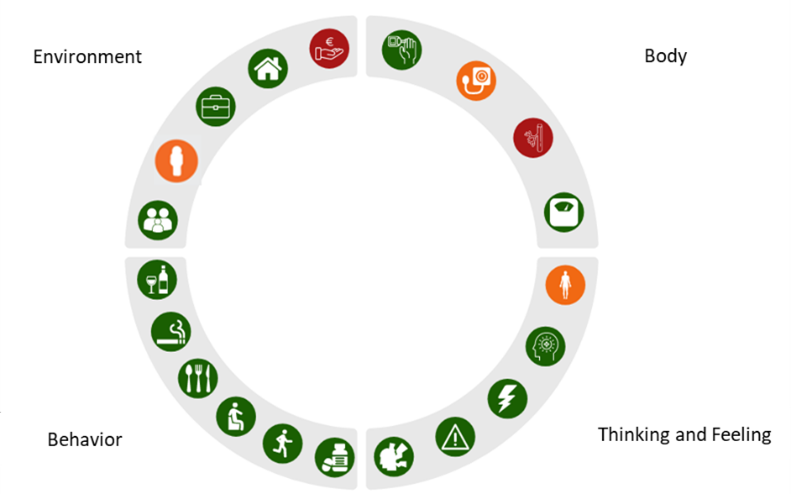
The profile wheel version 2.0, visualizing the results from the 360° diagnosis, consisting of questionnaires and measurements for four domains for people with type 2 diabetes in primary care.

We developed a 6-month intervention using a single-group design, which fits into a longitudinal care pathway due to its focus on a chronic disease. The intervention included the 360° diagnosis, tailored treatment, and both individual and group consultations (360° approach). The 360° diagnosis adopted a holistic approach, covering 4 domains: body, thinking and feeling, behavior, and environment. Each domain included specific parameters, underlying elements, and measurement instruments [10]. All data were integrated and visualized in a personal profile wheel (Figure 1), which displayed the scores for parameters in the 4 domains as colored icons—red, orange, or green—reflecting either a healthy status or areas for improvement. The NP, dietitian, and pharmacist used the profile wheel to discuss the results and determine the most suitable treatment in both a multidisciplinary meeting and an individual consultation with the patient. Additionally, patients were educated on T2D and related lifestyle factors, and shared their experiences with lifestyle changes during a group consultation attended by NPs, dietitians, and pharmacists. Changes over time were monitored through follow-ups at 3 and 6 months: the 360° diagnosis was repeated, and the results were discussed with the patient again.

We applied a mixed-method study design to assess the feasibility of the 360° approach in terms of acceptability, implementation, and potential effects within standard Dutch primary health care. At the end of the intervention, face-to-face semistructured interviews were conducted with both health care professionals and participants to evaluate the acceptability and implementation of the 360° approach. Additionally, the data collected from participants at baseline, 3 months, and 6 months as part of the 360° diagnosis were used to assess the potential effects of the 360° approach over time (ie, limited-efficacy testing).

### Participants

To participate in the study, 15 adults with T2D, a chronic disease, were recruited by 4 NPs from 2 Dutch primary care practices within a primary care cooperative in the Stevenshof area of Leiden, the Netherlands, where they were already receiving treatment for T2D. The participants were divided into 2 intervention groups, with 7 participants starting in March 2019 and 8 in May 2019, respectively, and the intervention ending in December 2019 and January 2020, respectively. Participants were eligible for inclusion in the intervention if they met the following criteria: (1) diagnosed with T2D, (2) on the verge of a change in their T2D treatment (eg, about to start another oral medication or an injectable drug), and (3) aged 30-80 years with a stable BMI between 25 and 40 kg/m^2^. Participants were excluded from the intervention if they met any of the following criteria: (1) undergoing dialysis, (2) receiving treatment from a psychiatrist, (3) in a palliative phase, (4) diagnosed with other diseases, or (5) unable to attend most meetings. In total, 7 professionals were involved in the intervention: 4 NPs, 1 dietitian (SB, a coauthor, was part of the research team), 1 pharmacist/project leader (AMKD, a coauthor, was part of the research team), and 1 employee from the medical diagnostic laboratory SCAL. In addition to the primary care professionals, experienced health care researchers from an applied research institute were part of the research team.

### Procedure

**Figure 2:**
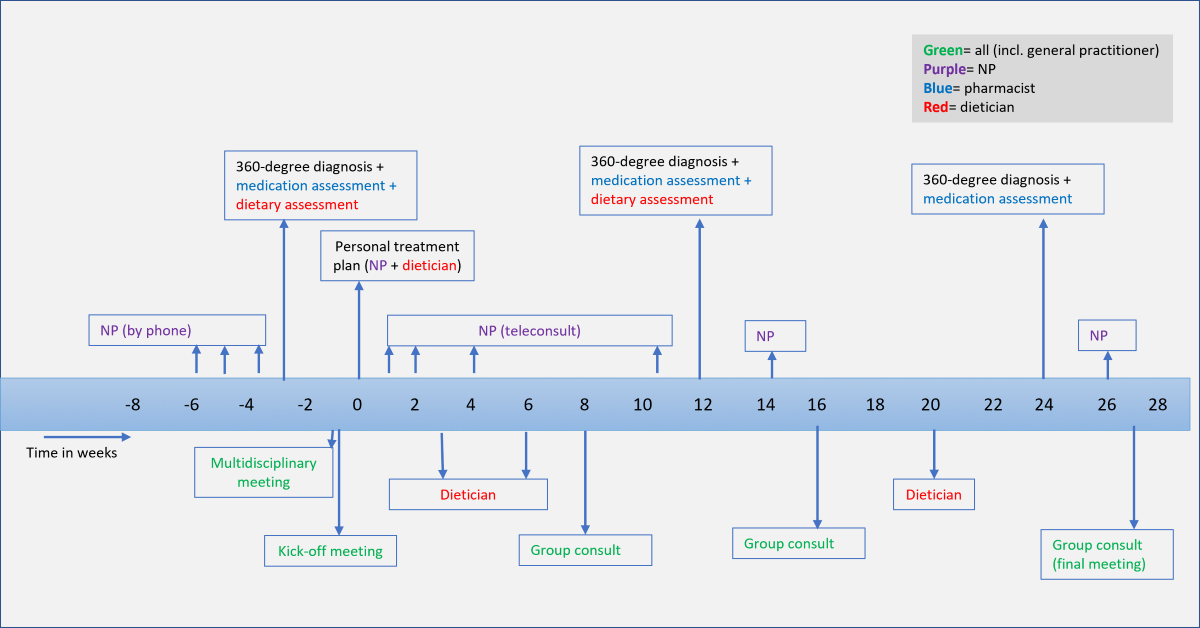
A visualization of the procedure of the 6-month intervention for people with type 2 diabetes in primary care which consisted of the 360° diagnosis, tailored treatment, and individual and group consultations (360° approach).

The procedures of the intervention are depicted in Figure 2. The 360° diagnosis was completed 3 weeks before the start of the intervention and consisted of clinical measurements for the body domain, performed by health care professionals, and surveys for the other 3 domains (health behavior, mental health, and socioeconomic environment), completed by the participants. Clinical measurements included clinical chemistry, blood pressure, anthropometric measurements, and an extended oral glucose tolerance test (OGTT). During the OGTT at SCAL (Leiden, the Netherlands), participants also had a medication review with the pharmacist and a dietary review with the dietitian. The clinical and survey data were then combined and visualized in a personal profile wheel. At the start of the intervention, the NP, pharmacist, GP, and dietitian discussed the results in a multidisciplinary meeting (using the profile wheel), which led to the creation of a personalized treatment plan for each participant, including advice on diet, physical activity, sleep, stress, and medication. The treatment plans were supervised by the GP. Following this, the NP (using the profile wheel) and dietitian reviewed the results and provided advice during individual consultations with the participants. Generally, the NP discussed and monitored the entire treatment plan, including medication, while the dietitian provided dietary advice during 3 consultations. The dietary advice included a 1-week very low-calorie vegetable diet, followed by the gradual reintroduction of protein and carbohydrates into the diet, personalized for each participant. Additionally, 3 face-to-face group consultations were organized for all participants with T2D, with NPs, the dietitian, and the pharmacist present. During these consultations, education on T2D and related lifestyle factors (ie, eating behavior and physical activity) was provided, and participants shared their experiences regarding lifestyle changes. After 3 and 6 months, the 360° diagnosis was repeated and visualized in an updated profile wheel, showing changes over time. A multidisciplinary team then reviewed whether the personal treatment plan needed adjustment based on the results. These results were discussed with the participants by the NP and dietitian. A summary of the results and advice based on the 360° diagnoses was emailed to the participants after each consultation in which the results were discussed. During the intervention, participants were offered glucometers to monitor how their bodies reacted to food intake. In addition to the scheduled consultations, participants and NPs maintained email and telephone contact based on each participant’s individual circumstances.

### Feasibility Study: Acceptability and Implementation

At the end of the intervention, face-to-face semistructured interviews were conducted with both health care professionals and participants. These interviews were audio-recorded, and both professionals and participants provided consent for the recording. All professionals, except 1 due to personal circumstances, were interviewed (n=6). At the final group consultation of the intervention, the 15 participants with T2D were asked to evaluate the intervention. One participant preferred not to be interviewed due to illness, and another did not attend the final group consultation. Thus, a total of 13 participants took part in the evaluation: 12 participants were interviewed, and 1 completed the questions in writing because it was logistically difficult to schedule an interview. The interviews with participants focused on their experiences with the following topics: (1) the start of the intervention, (2) the 360° diagnosis data collection, (3) the consultation meeting with the NP and dietitian, (4) the profile wheel, (5) the counseling during the intervention, (6) sustaining adherence to the intervention, and (7) the group meetings. The topic list for professionals included an additional topic: the collaboration between professionals and the multidisciplinary meeting. These topics specifically addressed the acceptability and implementation of the 360° diagnostic tool.

### Feasibility Study: Limited-Efficacy Testing

In total, as part of the 360° diagnosis, 14 participants completed the measurements at baseline, and at the 3- and 6-month follow-ups, while 1 participant completed the baseline and 3-month follow-ups and partially completed the 6-month follow-up. In this paper, we specifically report the effects over time for the parameters of mental health and lifestyle behavior, which represent the thinking and feeling domain and the behavior domain of the 360° diagnosis, respectively. For the parameters of the socioeconomic environment (environment domain), we include only the baseline results. The clinical and pathophysiological data and results for the body domain have already been published [12].

Quantitative data on the thinking and feeling, behavior, and environment domains were collected at baseline, and at the 3- and 6-month follow-ups as part of the 360° diagnosis [10]. The thinking and feeling domain included 5 measured parameters: perceived health (1 item from the Medical Outcomes Survey Short-Form 36 [13]), pain (1 item from the Medical Outcomes Survey Short-Form 36 [13]), mental health (WHO-Five Well-Being Index [14]), perceived stress (Perceived Stress Scale [15]), and problems with diabetes (Problem Areas in Diabetes Scale-5 [16]).

The behavior domain included 6 measured parameters: alcohol consumption (ie, the average number of glasses per day and binge drinking [17]), cigarette smoking (ie, number of cigarettes [18] and nicotine craving, Fagerström Test for Nicotine Dependence [19]), eating pattern (ie, fruit, vegetables, soda, large snacks, small snacks; questions based on Dutch dietary guidelines [20] and Dietary Guidelines for People with T2D [21], physical activity (Short Questionnaire to Assess Health-Enhancing Physical Activity [SQUASH] [22]), sedentary behavior (questions based on the Marshall Sitting Questionnaire [23]), and diabetes management (ie, glucose monitoring and medication adherence; revised Diabetes Self-Management Questionnaire [24]).

The environment domain was based on the Diagnostic and Statistical Manual of Mental Disorders, fourth edition [25] and the Dutch Self-Sufficiency Matrix [26], and included 5 measured parameters: family (ie, worries about children and relationships), loneliness, work, income, and housing (ie, neighborhood and residence).

### Data Analyses

The interviews were transcribed and analyzed using Word and Excel (Microsoft Corp.). We initially used a deductive approach, focusing on the main topics based on the procedures and aspects of the intervention (ie, the topics outlined in the interview guide and addressed in the semistructured interviews). Subsequently, the responses from participants with T2D and professionals were categorized into subcategories within these main topics using an inductive approach. Descriptive statistics were calculated for the 360° diagnosis using frequencies in the SPSS software package (version 29.0.2.0; IBM Corp.) for baseline, 3-month, and 6-month follow-up data on the parameters and underlying elements of the “thinking and feeling” and “behavior” domains. To test the potential effects over time of the 360° approach (ie, limited-efficacy testing), we conducted repeated measures analysis of variance in SPSS, with planned comparisons between measurement time points, applying a significance level of *P*<.05. For nonparametric variables, Cochran *Q* was calculated in SPSS to test changes over time. For the environment domain, only baseline descriptives were calculated using frequencies in SPSS.

## Results

### Baseline Characteristics of Participants With T2D

Table 1 presents the baseline characteristics of the 15 participants who completed the baseline measurements as part of the 360° diagnosis.

**Table 1:** Baseline characteristics for the 15 participants with type 2 diabetes who completed the baseline measurements as part of the 360° diagnosis.

Variable	Values
Men/women, n	10/5
Age (years), mean (SD)	59.6 (8.8)
Worries about children/no worries/no children, n	6/8/1
Worries about (lack of) relationship/no worries, n	3/12
Employed/not employed, n	10/5
Lonely/not lonely, n	1/14
Satisfied with residence/not satisfied, n	14/1
Satisfied with neighborhood/not satisfied, n	14/1
Worries about income/no worries, n	4/11
Years diagnosed with type 2 diabetes, mean (SD)	13.4 (5.2)
BMI (kg/m^2^), mean (SD)	34.1 (3.5)
Glycated hemoglobin (hemoglobin A_1c_) (mmol/mol), mean (SD)	67.6 (12.3)
Systolic blood pressure (mmHg), mean (SD)	136.7 (14.0)
Diastolic blood pressure (mmHg), mean (SD)	79.8 (7.1)

### Acceptability and Implementation of the 360° Diagnosis

#### Start of the Intervention

Participants reported a positive experience with the onboarding process of the intervention. Promoting factors included (1) the NPs proactively approached all participants for the intervention, (2) participants felt well-informed, and (3) participants were motivated to take part. The latter was influenced by the selection process, where professionals included individuals with T2D based on their motivation to participate. These participants were particularly motivated because they were facing complicated or challenging situations: (1) managing T2D, such as maintaining blood glucose levels within range, and (2) being on the verge of starting insulin injections. Participants identified their main motivations for participating as (1) losing weight, (2) discontinuing insulin injections, or (3) avoiding the need to start insulin injections. One participant said, “Either you have this choice, to participate in the project [read: intervention] or you don’t and you’re out of luck. Then you have to start injecting. That's your only alternative. So yes, harsh, but fair.” Participants expressed concerns about (1) the amount of information provided at the start, and (2) insufficient details about the vegetable diet. They also suggested that the intervention should begin for all participants at the same time, including the vegetable diet.

#### Data Collection for the 360° Diagnosis

Overall, participants were very satisfied with the 360° diagnosis, including completing the questionnaires. However, some participants noted that the 360° diagnosis was intensive, with the questionnaire being lengthy, containing redundant questions, and not always aligning with their personal circumstances. Professionals identified the internet connection on the tablets and participants’ computer skills as hindering factors.

#### Advice/Consult Meeting With the NP and Dietician

Participants found the discussion of the treatment plan during the first individual consultation to be positive. Most participants noted that the professionals provided (urgent) advice, but emphasized that they themselves were responsible for its implementation. The motivation to adhere to and actually implement the advice over time appeared to be influenced by (1) favorable results (such as improvements in weight and blood glucose values), (2) support from the social environment and professionals, and (3) dietary variation. One participant said with regard to the latter: “I have to say: I’ve also become more creative, more creative with food, so really looking for flavor. I mean...Vegetable diet...Everything tastes like warm cucumber at one point. Lettuce, soup, pepper, cucumber, you name it,..., a bit of rocket to add a little spice. It’s not like: ‘We'll just throw a nice dressing on top’. You have to try to get flavor into something.” Overall, professionals noted that participants were motivated by and satisfied with the first individual consultation, which discussed the treatment plan. Professionals highlighted the following promoting factors: (1) insight gained by participants (eg, the effect of lifestyle on blood sugar levels) and by professionals (eg, reducing insulin levels through diet in people with T2D), (2) involvement of the participants’ direct social environment (eg, partners, children) in the consultations, and (3) positive feedback provided by professionals to participants. Professionals also identified difficulties in implementing and sustaining advice on physical activity and the vegetable diet as barriers to success.

As a point of attention, participants suggested scheduling the discussion of the treatment plan earlier, ideally directly after the OGTT, to discuss the results more promptly. However, this was practically challenging due to the need to wait for the OGTT results. Professionals identified 2 key points for attention: (1) the underlying causes of participants’ issues, such as environmental factors and stress, should be properly addressed, and (2) professionals should simplify the explanation of the intervention for participants. A professional said of the latter “I often find myself looking at it like, does it land with the patients? I notice that we still need to make a translation to explain it simply. These are people who are not very highly educated, and even if they are highly educated, it is an area they are not familiar with. Then you really have to explain it in lay language.”

#### The Profile Wheel

The profile wheel was reviewed with all participants during the discussion of the treatment plan in the first session. They were encouraged to view the profile wheel at home by logging in. One participant did so: “I found it very impressive and it's actually a trigger of these are my data and look what an improvement there is....Now you could just look back at that, at home. I found that useful.” Most participants did not view the profile wheel at home because it had already been explained during the counseling session, it required computer knowledge, or they were too busy with other commitments. Professionals indicated that while the profile wheel is helpful for them (eg, in discussing their own findings and topics more easily), it is also complex for participants. Professionals noted the absence of sleep as a topic. They also identified areas for improvement, such as emphasizing the value of reviewing the profile wheel at home for participants and providing training for professionals to better explain and justify the advice.

#### Counseling During the Intervention

Both participants and professionals viewed the contact between them positively, mainly due to the ease of communication and the intensive guidance provided. A professional indicated that there was a shared enthusiasm: “There were all kinds of people who had tried something. You just noticed with everyone that the results were good and everyone became very enthusiastic. It became easier. And just scheduling an appointment also felt much easier.” Participants also appreciated the continuity of professionals, and the meetings with the NP, dietician, and pharmacist: “It is also nice that you always deal with the same NP.”; “The dietician can also understand that sometimes things also go completely wrong. Yes, really a pillar of support.” The conversation with the pharmacist was valuable, as participants received advice on topics such as side effects, which they do not typically get in a standard consultation. Professionals also appreciated the low-threshold communication, cooperation, and enthusiasm among the team, which fostered greater mutual coordination. One professional said, “Yes, it’s very nice because you worked together much more intensively. It’s nice to do a consultation together. Then you have more insight into things together.”

Points of concern raised by professionals included (1) the time-consuming nature of counseling and (2) some participants experiencing negative side effects from the lifestyle intervention, such as temporary vision reduction. To prevent such side effects, one professional suggested more stringent monitoring, such as scheduling an eye scan at the start of the intervention for participants who had not recently had one. “Yes, that we thought of if you are going to start insulin, because that will also cause the sugar to drop quickly, have those people take an eye picture. We haven’t done that in anyone now.” Furthermore, professionals suggested increasing the involvement of the GP and physiotherapist in the intervention.

#### Sustained Intervention Adherence

Participants reported that they developed a healthier lifestyle and gained more knowledge about T2D, among other benefits. One participant indicated, “A healthier lifestyle. Changed my view on food. Healthier, better, low-carb. And that I am still susceptible or a bit in the danger zone for diabetes. And that I just have to keep a very close eye on that. I was quite fond of wine with dinner. And gee a beer what does it matter. I found out that it does matter. So I scaled that back quite a bit....Because diabetes that was always something of you know it exists, but oh I don't suffer from it myself...” Other promoting factors for sustaining the intervention were social support from the environment (eg, a partner or other participants), a positive attitude, low-threshold contact, continuous guidance from the professionals, and experiencing positive effects (such as injecting less insulin or losing weight). Professionals indicated that a key promoting factor for sustained adherence on their part was having an ultimate manager or someone in charge who provided support: “If she had not taken on so much organization, things would have been different. She took almost all the work off our hands. We were really just there for the patients.”

#### Group Meetings

Participants were generally positive about their experience with the group meetings. They highlighted the added value of these sessions and enjoyed exchanging experiences with others. One participant indicated, “Those [read: group meetings] were great. You learn from each other that this is like this and that is like that and one does it that way and the other that way. They also included food and certain things they used. Yes you learn quite a bit from each other.” Besides the group meetings, a WhatsApp group was created to facilitate sharing experiences, motivating each other, and complementing each other between the meetings. This WhatsApp group, intended for interaction and moderated by a professional, was not widely used by the group members. However, professionals found the group meetings valuable, noting that they allowed participants to exchange experiences, motivate one another, offer recognition, and share helpful tips. One professional indicated, “And you did see that those were just very valuable meetings and that everyone enjoyed sharing and...that of course they [read: participants] do need to be challenged a bit, but that they also found it really valuable to participate in such a group meeting, to be there. And the health care professional who ran the meeting also made it really fun, including the last session with a kind of quiz, with all kinds of fun questions about things we think everyone knows but we didn’t really know ourselves either, in terms of food products and things like that.” Another professional said, “...Those group consultations were great fun, because people knew how to motivate each other and provide tips. So I do believe in the power of group consultations. I do think that's a very nice way. And there is relatively little preparation for the caregivers.”

Participants suggested 2 points for attention: (1) scheduling the meetings at times that are more convenient for working participants, and (2) avoiding jargon language used by professionals. Professionals recommended considering an increase in the frequency of the group meetings to further enhance participant engagement and support: “So group consultations for me could be more frequent and earlier in the process. I think that’s good for group formation.” Additionally, professionals suggested incorporating activities such as cooking together or reading labels as part of the group meetings to make them more interactive and practical for participants. One professional said, “And maybe occasionally a meeting of, cooking together or something like that. Something like, what are the possibilities? How can you make a meal tasty? Some people can cook well and they play with food. But other people can’t get beyond making a vegetable soup. That does get a bit boring at some point. I myself am very much into inventing things and new things, but if you don’t have that much...People are very different in this.”

### Limited Efficacy Testing of the 360° Approach

In Table 2, the effects over time are shown for the surveys related to the “thinking and feeling” and “behavior” domains. For the thinking and feeling domain, the scores for mental health increased (∆*M*=7.20; *P*=.02) and the scores for problems with diabetes decreased (∆*M*=2.07; *P*=.01) significantly between baseline and 3-month follow-up. However, these changes were not maintained after 6 months. After the 6-month follow-up, the scores for perceived stress significantly decreased (*∆M*=0.19; *P*=.04). No significant changes over time were observed for perceived health (*P*=.16) and pain (*P*=.95). For the behavior domain, changes were seen for fruit and large snack intake. The scores for fruit intake decreased significantly between baseline and 3-month follow-up (∆*M*=1.09; *P*=.01), and this change was maintained after the 6-month follow-up (∆*M*=0.96; *P*=.02). Large snack intake showed a decreasing trend over time, caused by a large decrease after 3 months (*F*_1,12_=16.94; *P*=.001). No changes were seen over time for other food groups (ie, vegetables [*P*=.17], small snacks [*P*=.11], and soda [*P*=.65]), alcohol consumption (*P*=.22), and binge drinking (*P*=.07). For smoking behavior, significance tests were not applicable because nobody smoked. For physical activity (measured with the SQUASH) and sedentary behavior (measured with the Marshall Sitting Questionnaire), the data were of insufficient quality for analysis as a result of experienced difficulties in completing the questionnaires by participants.

**Table 2:** Mean (sub)scores, percentages, and changes over time (*P* value) for the surveys related to the “thinking and feeling” and “behavior” domain of the 360° diagnostic tool as filled in by participants with type 2 diabetes^a,b^.

Domain, parameters, and elements					Scale extremes (n)		Baseline		3 months		6 months		Overall *P* value
**Thinking and feeling**													
	Perceived health			1-5 (15)		2.40 (0.74)		2.07 (0.70)		2.20 (0.68)		.16	
	Pain			0-5 (15)		2.00 (1.73)		2.13 (1.89)		2.07 (1.58)		.95	
	Mental health			0-100 (15)		66.93^y^ (21.46)		74.13^z^ (15.18)		70.93^y,z^ (13.13)		.04	
	Perceived stress			0-4 (15)		1.75^y^ (0.33)		1.59^y,z^ (0.58)		1.56^z^ (0.48)		.13	
	Problems with diabetes			0-4 (15)		3.60^y^ (3.64)		1.53^z^ (3.11)		2.07^y,z^ (3.24)		.05	
**Behavior**													
	**Alcohol consumption**												
		Glasses/day		0-8 (14)		0.97 (1.49)		0.25 (0.38)		0.43 (0.53)		.21	
		**Binge drinking**		N/A^c^ (15)		N/A		N/A		N/A		.07	
			Once or more	N/A		8/15 (53)		5/15 (33)		4/15 (27)		N/A	
			Never	N/A		7/15 (47)		10/15 (67)		11/15 (73)		N/A	
	**Cigarette smoking**												
		Number of cigarettes		N/A (15)		0		0		0		NA	
		Craving		N/A (15)		0		0		0		NA	
	**Eating pattern**												
		Fruit (pieces/day)		0.04-6 (11)		1.31^y^ (1.11)		0.22^z^ (0.32)		0.35^z^ (0.44)		.04	
		Vegetables (serving spoons/day)		0.04-6 (15)		2.90 (1.27)		3.68 (1.57)		3.54 (1.94)		.17	
		Soda (glasses/day)		0 to 4 (12)		0.24 (0.11)		0.27 (0.08)		0.27 (0.08)		.65	
		Large snacks (fast-food, pastries; pieces/week)		0.25-10 (13)		0.72^y^ (0.27)		0.39^z^ (0.25)		0.65^y,z^ (0.90)		.001	
		Small snacks (cooky, candy, chips; pieces/day)		0.1-4 (13)		0.54 (0.47)		0.23 (0.16)		0.31 (0.57)		.11	
	**Diabetes management**												
		Glucose monitoring (%yes)		N/A (15)		8/15 (53)		12/15 (80)		11/15 (73)		.16	
		Medication (%yes)		N/A (15)		15/15 (100)		14/15 (93)		12/15 (80)		.17	
		Forget medication (%yes)		N/A (11)		3/11 (27)		2/11 (18)		2/11 (18)		.72^y^	

^a^Different superscripts (y, z) in a row mean a significant difference (*P*<.05). Furthermore, analysis of variance was primarily used for statistical analysis. However, for the elements binge drinking, glucose monitoring, medication, and forget medication, which were measured dichotomously, a Cochran *Q* test was performed.

^b^Data for baseline, 3 months, and 6 months are presented as mean (SD) or n/N (%).

^c^N/A: not applicable.

## Discussion

### Principal Findings

The aim of this paper was to evaluate the feasibility in terms of acceptability, implementation, and efficacy of the 360° approach in standard primary health care. First, the results of the semistructured interviews showed that participants and health care professionals were overall positive about the 360° approach, including aspects regarding onboarding, data collection for the 360° diagnosis, advice/consultations with the NP and dietician, the profile wheel, counseling during the intervention (including the collaboration among the professionals), and the group meetings. Second, the results of the semistructured interviews also showed that participants and health care professionals indicated promoting and hindering factors regarding the implementation of the 360° approach. Promoting factors were (1) the care, attention, support, and experience of professionals; (2) the multidisciplinary team; (3) the social support; and (4) experiencing positive health effects. Hindering factors were (1) too much information, (2) survey-related issues, and (3) time-consuming counseling. Finally, regarding the potential effects of the 360° approach over time, improvements were observed at 3 months in mental health, problems with diabetes, and fast-food consumption. At 6 months, there was a reduction in perceived stress and fast-food consumption. Additionally, fruit intake decreased at both 3 and 6 months, likely due to the focus on a low-carbohydrate diet in the personalized treatment plan for most participants.

Our results show that the 360° approach promotes a patient-centered model, with several key components supporting this approach and being crucial for its application in standard primary health care [27]: (1) selection and onboarding; (2) holistic approach; (3) multidisciplinary approach; (4) specific and tailored attention, communication, and support; and (5) health promotion and positive health effects. We will discuss each of these components below.

The first component is the selection and onboarding of motivated patients with T2D. The NPs identified and specifically selected patients who were motivated to change their behavior. This motivation stemmed from the negative consequences of T2D that these patients were experiencing. A patient’s motivation is a crucial prerequisite for participating in the 360° diagnosis and subsequent tailored treatment. The importance of motivation and intention in behavior change is emphasized by several theoretical models, such as the Transtheoretical Model [28] and the Theory of Planned Behavior [29]. Therefore, the approach will differ for patients with T2D who are motivated versus those who are not motivated. The approach for patients with T2D who are not motivated to change their behavior will focus on increasing awareness of the negative consequences if their behavior is not changed, while emphasizing the benefits of making those changes [28]. Additionally, onboarding plays a crucial role in ensuring that patients are well-informed and know what to expect, which may help enhance their motivation.

The second component is the use of a holistic approach, which allows treatment to be tailored to individual circumstances. This approach was facilitated through the personal 360° diagnosis and profile wheel, which highlighted areas for improvement across the body, thinking and feeling, behavior, and environment domains. The 360° diagnosis and profile wheel were helpful for both the patient and the professional by identifying areas for improvement, potentially contributing to the empowerment of the patient.

The third component of a person-centered approach is the incorporation of a multidisciplinary strategy, where multiple health care professionals (eg, GPs, NPs, dieticians, and pharmacists) collaborate to develop a cohesive treatment plan. This cooperation fostered mutual coordination and enthusiasm, facilitated by low-threshold contact among the professionals. Additionally, the presence of a multidisciplinary team enhances the holistic approach. Involving additional health care professionals, such as a physiotherapist, would provide further value for individuals with T2D. Previous studies have shown that a multidisciplinary approach, where diverse health care teams are involved, improves patient outcomes, including glycated hemoglobin (HbA_1c_), blood pressure, and blood lipid levels, and has also been found to be cost-effective [8].

The fourth component of a person-centered approach is the specific and tailored attention, communication, and support provided to each individual through continuous interaction with health care professionals (NPs, dieticians, and pharmacists) and social support from partners and group members. This involvement and support are crucial for adhering to the treatment plan. In the 360° approach, health care professionals frequently checked in with participants about their progress, which made the participants feel truly cared for. Social support was provided through the involvement of partners, who were invited to individual consultations, and through group meetings with fellow participants. The support from health care professionals is considered a key component of the 360° approach. Social support plays an essential role in helping participants sustain compliance with the intervention, although a group approach must meet certain preconditions to establish and maintain safety, trust, and cohesion within the group [30]. Additionally, it may not be suitable for everyone, as it depends on the individual’s preferences and needs.

A key component of a person-centered approach is its focus on health promotion and positive health outcomes. Patients with T2D were motivated to follow the treatment plan to improve body weight and blood glucose levels. They reported experiencing positive health effects from the 360° approach. De Hoogh et al [12] described these positive effects, including improvements in body weight, waist-to-hip ratio, triglyceride levels, HbA_1c_, fasting glucose, and 2-hour glucose levels after 3 and 6 months. However, the positive changes were most pronounced after 3 months and slightly decreased after 6 months. This suggests that sustaining improvements in the longer term (6 months) may be more challenging. One possible explanation for the positive short-term health effects is that the 360° approach may have facilitated more effective shared decision-making and encouraged the identification and setting of realistic goals [31,32].

Our study findings suggest that the 360° approach (1) is acceptable, as both people with T2D and health care professionals responded positively to it; (2) is implementable in standard primary health care, with areas for improvement identified; and (3) has the potential to foster positive health changes in people with T2D, including improvements in mental health, stress levels, diabetes-related issues, and fast-food consumption. Additionally, the study highlighted several ways in which the 360° approach could be improved in standard primary health care. First, it would be beneficial to have someone designated to organize the multidisciplinary team, allowing the professionals involved to focus primarily on the patient. In our study, 1 health care professional voluntarily took on this management role within the team. Second, the professionals found counseling to be time-consuming, which could pose a challenge in primary care settings where time constraints and staff shortages are already a concern. Third, while the 360° approach can support NPs and other professionals in primary care, it may not be suitable for all patients with T2D, particularly those who are not motivated. As a result, professionals could consider selecting patients who are motivated to participate. Finally, the 360° diagnostic tool could be further improved by better supporting people with T2D who have limited or no computer skills, or reduced vision. Additionally, shortening the questionnaires required for the 360° diagnosis would enhance its usability.

### Limitations and Strengths

Some limitations should be noted. The small study population (n=15) and the limited number of primary care practices involved (n=2) restrict the generalizability of the results. The primary focus of this feasibility study was implementation (ie, determining whether the 360° approach could be implemented as planned). Because of the use of a convenience sample, a short follow-up period, limited statistical power, and the lack of a control group, our insight into the effects of the 360° approach on the underlying elements of the domains [11] is limited. Future research is needed to assess the efficacy, cost-effectiveness, scalability, and long-term effects of the intervention. Additionally, some validated scales and instruments, such as the SQUASH (which measures physical activity) and the Marshall Sitting Questionnaire (which measures sedentary behavior), proved to be complex and difficult for participants to complete. Therefore, even if scales have good psychometric properties (eg, reliability and validity), they may not be suitable for use in a tool like the 360° diagnosis. Therefore, these scales and instruments need to be adapted or replaced—perhaps with wearable technology to monitor individuals’ health and exercise—in the further development of the 360° diagnostic tool to enhance its user-friendliness. Nevertheless, this study also has several strengths, including (1) its implementation in regular primary care, (2) the multidisciplinary approach, and (3) high adherence to advice (especially during the first 3 months).

### Conclusions and Future Directions

Our findings suggest that the 360° approach is acceptable to both people with T2D and health care professionals, implementable in standard primary health care, and potentially effective in fostering positive health changes among people with T2D. The 360° diagnosis serves as a tool for professionals to provide patient-centered care. It can facilitate shared decision-making between patients and health care professionals, promote multidisciplinary collaboration, and support and empower both patients and professionals, leading to positive health outcomes for the patient. The results suggest that implementing the 360° diagnosis and subsequent tailored treatment in standard primary health care is feasible. Therefore, there is a window of opportunity for the adoption of the 360° diagnostic tool and tailored treatment in primary care settings. Future research should focus on examining the scalability and long-term effects of the intervention. However, the use of the OGTT in a laboratory, the 360° diagnosis and profile wheel, and the additional time investment by health care professionals fall outside standard primary care practice. These factors may affect the applicability and scalability of the 360° approach in other primary care settings in the Netherlands. Additionally, the 360° diagnostic tool was initially developed specifically for people with T2D in the Netherlands, but it could also be adapted for use in other countries, for different diseases (such as chronic conditions), and for various purposes, including prevention or new diagnostic applications (eg, in pregnancy monitoring), as well as other health care settings [10].

## Abbreviations

GP: general practitioner
HbA1c: glycated hemoglobin
NP: nurse practitioner
OGTT: oral glucose tolerance test
SQUASH: Short Questionnaire to Assess Health-Enhancing Physical Activity
T2D: type 2 diabetes
WHO: World Health Organization

## Data Availability

The data sets generated and analyzed during this study are available from the corresponding author upon reasonable request.
